# The Parasitoid, *Verticia fasciventris* Causes Morphological and Behavioral Changes in Infected Soldiers of the Fungus-Growing Termite, *Macrotermes carbonarius*

**DOI:** 10.1673/031.011.4701

**Published:** 2011-04-11

**Authors:** Kok-Boon Neoh, Chow-Yang Lee

**Affiliations:** Urban Entomology Laboratory, Vector Control Research Unit, School of Biological Sciences, Universiti Sains Malaysia, Minden, Penang, 11800, Malaysia

**Keywords:** aggression behavior, blowfly, Calliphoridae, parasitism

## Abstract

The larval parasitoid *Verticia fasciventris* Malloch (Diptera: Calliphoridae) develops in the head of soldiers of the fungus-growing termite *Macrotermes carbonarius* (Hagen) (Isoptera: Termitidae). Morphological and behavioral changes in the host were evaluated and the termite castes and stages that were parasitized were identified. The larval emergence process is also described and possible mechanisms for the parasitoid fly's entry into the host body are discussed based on qualitative observations. Only a single larva per host was found. The mature larva pupated outside the host's body by exiting between the abdominal cerci. Parasitized soldiers possess a short and square-shaped head capsule, a pair of notably short mandibles, and a pair of 18-segmented antennae. Although parasitized soldiers were statistically less aggressive than healthy soldiers (P < 0.05), they expressed varying levels of aggression. Both minor and major soldiers can be parasitized and based on evidence from presoldiers, parasitization may begin during the precursor stages of soldiers. However, the stage at which parasitism first occurs has not been determined.

## Introduction

Interactions between immature parasitoids and hosts often cause drastic changes in the host. These changes can affect both individual and colony-level responses in social insects. Parasitism in termites leads to various costs for host colonies. For instance, infected workers often have reduced fitness. This may alter foraging rates, which would result in less energy (= food source) channeling to the young swarmers, thereby decreasing a colony's reproductive output (Fuller and Jeyasingh 2004). In the Caribbean termite, *Nasutitermes acajutlae*, infected termites have higher predation risk by lizard predators (Fuller et al. 2003). Parasites also cause behavioral changes in hosts (O'Donnell 1997). For example, Korb and Fuchs (2006) found that compared to non-infected colonies, mite-infected drywood termites performed more feeding and resting rather than the allogrooming and proctodeal trophallaxis that can prevent parasites being transmitted within the host colony. Furthermore parasitism of termites, which are known to be ecosystem engineers (Dangerfield et al. 1998), may have a large impact on ecosystem processes (Thomas et al. 1999).

*Macrotermes carbonarius* (Hagen) (Isoptera: Termitidae) is an abundant fungus-culturing termite species in Southeast Asia. It is found mainly in Thailand, Cambodia, Vietnam, the Malay Peninsula, and Borneo (Roonwal 1970). *M. carbonarius* exhibits a forked developmental pathway, in which the developmental lines of workers and nymphs split at the first molt. This species exhibits size dimorphism in the soldier castes, with head widths of 4.40 and 2.95 mm for the major and minor soldiers, respectively. The soldiers differentiate from the worker line: major soldiers develop from fourth larval instars, and minor soldiers derive from third larval instars, and both pass through the presoldier stage (Neoh and Lee 2009a). Presoldiers are non-sclerotized and possess soldier-like features (e.g. mandible, head capsule). *M. carbonarius* usually forages for food on open ground (Inoue et al. 2001). Soldiers often stand in a line at both sides of the foraging processional columns and defend the workers. (Sugio 1995). Because of this behavior, soldiers may be at risk of predation and of parasitism.

Life histories are exceptionally diverse among blowflies (Oldroyd 1964). For example, blowfly larvae that cause myiasis are of medical importance (Askew 1971), whereas others are carrion feeders and protelean parasites of vertebrates and invertebrates (Oldroyd 1964; Colyer and Hammond 1968). The dipteran genus *Verticia* of the subfamily Bengaliinae is widely distributed in the Malay Peninsula, Borneo, Singapore, and Thailand (Tumrasvin et al. 1979; Kurahashi et al. 1997). *Verticia fasciventris* Malloch (Diptera: Calliphoridae) is a small-sized fly with a body length of 5.5 mm for males. The fly is brownish-yellow in color with three indistinct longitudinal stripes on the thorax (Tumrasvin et al. 1979). Little is known about the biology of this fly, but it is known to parasitize some species of *Macrotermes* (Sze et al. 2008).

Parasites have significant evolutionary effects on their hosts, especially on social behavior. Therefore, a better understanding of the relationships between social insects and their parasites is necessary to elucidate the adaptive strategies of both hosts and parasites. In the present study, we quantified the effects of the fly parasitoid *V. fasciventris* on both morphological and behavioral changes in the termite host *M. carbonarius*. We also examined the prevalence of parasitism in this host and the process of fly larva emergence. Based on our qualitative observations, we discuss possible mechanisms whereby fly parasitoid enters the termite host's body.

## Materials and Methods

### Study area

The study was conducted at Penang Island, located on the northeastern coast of the Malaysian Peninsula, between January 2008 and April 2009. Parasitized termite mounds were surveyed at two locations: (1) Minden Campus of Universiti Sains Malaysia (USM) (5° 21′ N and 100° 18′ E): the campus encompasses an area of approximately 100 ha, and the density of *M. carbonarius* mounds on the campus can exceed 3 mounds/ha; and (2) Bayan Lepas (5° 17′N and 100° 15′E): about 3 km on each side of the road along Jalan (Road) Tun Dr Awang. The sites were approximately 8 km apart.

**Figure 1.**
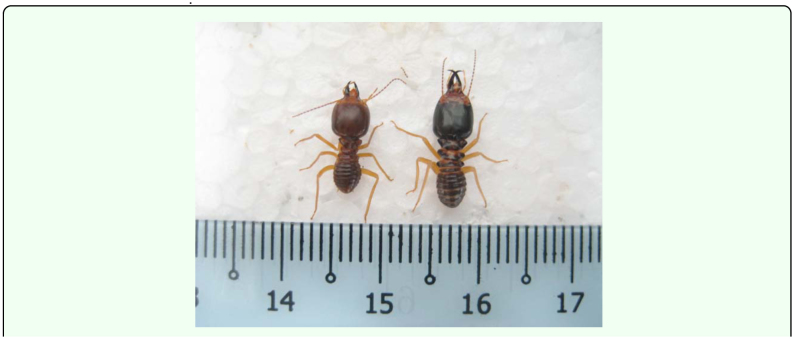
*Macrotermes carbonarius* soldier. Left: parasitized soldier; Right: healthy soldier, (Scale bar = 1.0 mm). High quality figures are available online.

Termite mounds were broken up by removing their outer wall. Under the wall, the colony was exposed to reveal fungus combs and clay pillars. The colonies were surveyed for the presence of parasitized soldiers as these soldiers tended to aggregate at the exposed part of the nest. Parasitized soldiers and presoldiers, which were recognizable by their conspicuously square shaped head capsule and a pair of short mandibles (Figure 1), were collected and transported to the laboratory. Meanwhile, samples of healthy soldiers were also collected for statistical analysis. Samples were placed in plastic storage boxes (320 mm × 250 mm × 130 mm) containing moistened vermiculite. They were kept at a constant temperature (28 ± 1° C) and relative humidity of approximately 90%. The infected soldiers were kept along with samples of fungus comb and major and minor workers to ensure that the soldiers were fed.

### Emergence of larval parasitoid from host

Host and larval parasitoid behavior before and after emergence of the larval parasitoid were video recorded (Sony Digital Handycam 8, www.sony.com). The larvae and adult flies that emerged were preserved in 70% alcohol and slide mounted for species identification.

### Morphometric analysis

Random samples of infected and healthy soldiers were taken and preserved in 70% alcohol for morphometric analysis. Various parts of parasitized soldiers (Neoh and Lee 2009a) were observed under an Olympus SZ61 stereo microscope with IC Imaging Standard, version 2.1 (Olympus, www.olympus.com), and measured by using Analysis® Image Processing Software (Soft Imaging System GmbH, www.softimaging.net). The following measurements of infected soldiers were taken: width of the head and pronotum at the widest point; length of the head at the side base of the mandibles; length of the antenna; and length of the third hind tibia. Discriminant analysis was performed on the measurements to determine which soldier castes were parasitized (using SPSS Version. 11.0, SPSS Inc., Chicago, IL). The Student T-test also was used to compare the morphological variation between parasitized and healthy soldiers.

The heads of 10 parasitized soldiers and 5 parasitized pre-soldiers were dissected soon after transport to the laboratory to investigate the termite's head content. Ten workers each from eight mounds and 20 late larval instars [third larval instars (L3) and fourth larval instars (L4), respectively] from two mounds at the USM also were randomly taken and inspected for the presence of larval parasitoids.

### Agonistic test

The behavior of parasitized *M. carbonarius* soldiers also was examined. A parasitized *M. carbonarius* soldier was placed in a Petri dish (50 mm diameter) to confront a healthy *Macrotermes gilvus* (Hagen) soldier (a sibling species that is dominant in Southeast Asia; these two species often compete and fight in nature). The experiment was run 10 times with 10 different individuals of each species. Confrontations between healthy soldiers of both species were used as the control. Confrontations were video recorded for five minutes. The aggression behaviors were recorded using the following definitions (Jmhasly and Leuthold 1999): (1) examination or antennation (contact between antennae or between antennae and body); (2) alarm or avoidance in the form of jerking (a repeated rapid body movement that moves the body front and back) and chasing or escaping; and (3) aggression (mandibles open, seizing or biting). The behaviors were recorded at 10 s intervals (thus, 30 observations for each pair).

## Results

### Dipteran parasitoid infestation rates

The rate of parasitism differed significantly between the two study sites. Seven out of 23 examined mounds (30.4%) at the USM site contained parasitized soldiers whereas at Bayan Lepas only 1 out of 13 colonies (7.7%) was parasitized.

### Emergence of larval parasitoid from host

**Figure 2.**
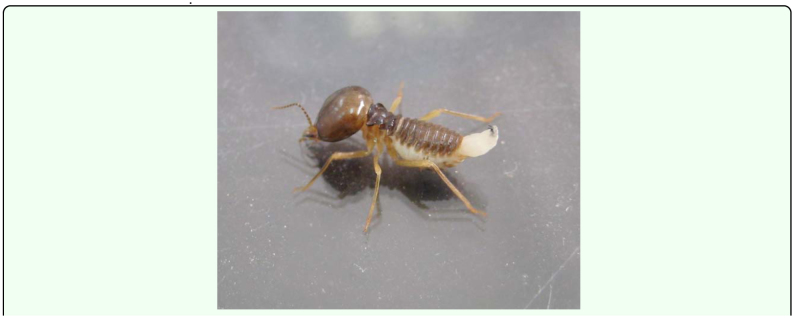
Larval parasitoid emergence posture in a parasitized soldier. High quality figures are available online.

A total of 15 larval emergences were observed and video recorded during the study. Only a single larva per host was found. The mature larva moved through the host from the head through the body and then exited between the abdominal cerci using mouth hooks. The mouth hooks always faced upward (Figure 2), and the larva contracted intensively during the departure. The process took about 10 minutes to complete. The emerging larva retreated into the host body when disturbed. The host usually stood still to allow the larva to depart from its body. No aggressive behavior was observed between the newly emerged larva and other termite individuals. The larva burrowed into the vermiculite and buried itself for pupation. The hosts continued with their routine as normal soldiers for another 1–2 days under laboratory conditions before dying. The pupae took approximately 12–13 days to develop to the adult stage under laboratory conditions. The fly was identified as *V. fasciventris* by Dr. H. Kurahashi (International Department of Dipterology, Tokyo, Japan).

### Morphological and behavioral alterations of host

Parasitized soldiers could be readily recognized by the presence of a short- and square-shaped head capsule and a pair of notably short mandibles relative to healthy soldiers (P < 0.001) (Table 1). The head capsule is thick and almost square in shape and broader at the middle of the head. The newly formed parasitized soldiers were less pigmented, with a lighter brown head capsule and body, compared to healthy soldiers.

**Table 1.**
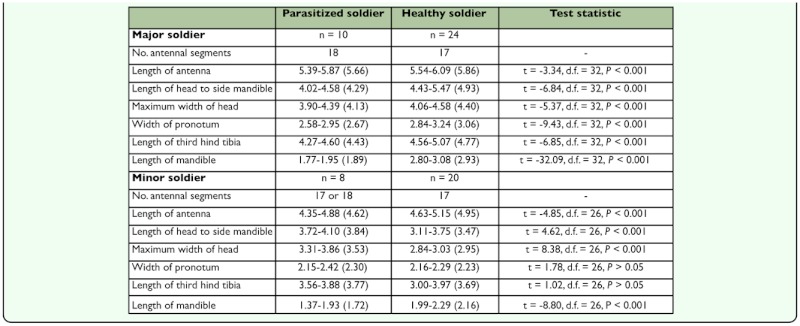
Range and mean (in parentheses) of measurements in individuals of the parasitized soldiers and healthy soldiers of *Macrotermes carbonarius*.

Healthy soldiers possess 17-segmented antennae, and the third segment is 2 times longer than the second segment in major soldiers and 1.5 times longer in minor ones. In most cases, parasitized soldiers had 18 well-defined antennal segments, and the third segment was sub-equal to the second segment.

The head of healthy major soldiers was significantly larger than that of parasitized major soldiers (P < 0.001) (Table 1). In contrast, parasitized minor soldiers had a larger head capsule and shorter antennae (despite having an additional antennal segment) (P < 0.001) compared to healthy minor soldiers; however, their pronotum width and tibial length were not significantly different (P > 0.05) (Table 1).

**Table 2.**
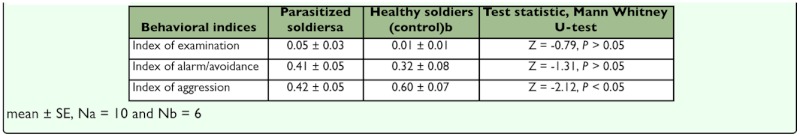
Aggression behaviour indices of parasitized soldiers and healthy soldiers of *Macrotermes carbonarius* in response to *Macrotermes gilvus*.

Table 2 shows the behavioral indices of parasitized soldiers and healthy soldiers of *M. carbonarius* with *M. gilvus*. Three general behaviors were observed in the parasitized soldiers. First, in several cases parasitized soldiers inspected the alien species *M. gilvus* and then walked away. Second, a series of jerkings by parasitized soldiers occurred after contacting *M. gilvus*. In most instances, parasitized soldiers retreated and fled from attack by *M. gilvus*. Third, parasitized soldiers shook their heads vigorously towards *M. gilvus* with mandibles open. However, the short deformed mandibles rendered parasitized soldiers incompetent fighters in any agonistic interaction, and parasitized soldiers suffered high mortality (in 8 out of 10 replicates the parasitized soldiers were killed during the interaction). Healthy soldiers were significantly (P < 0.05) more aggressive than parasitized soldiers (Table 2).

**Figure 3.**
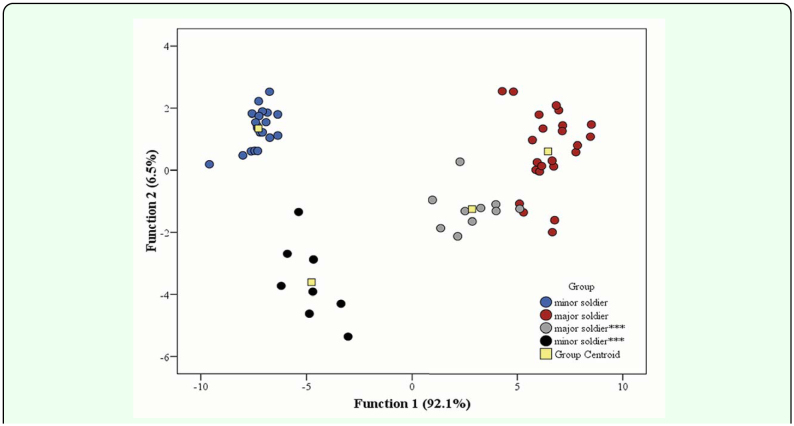
Canonical discriminant functions of *Macrotermes carbonarius* soldiers. ***: parasitized soldier. High quality figures are available online.

### Susceptibility of host caste to parasitization

The discriminant analysis easily divided the examined termites into parasitized and healthy soldiers (Figure 3). The first function separated minor soldiers from major soldiers, which accounted for 92.1% of the total variance (eigenvalue: 40.046) and was heavily weighted on head width, antenna, and pronotal length. The second function, which accounted for 6.5% of the total variance (eigenvalue: 2.834), isolated the parasitized soldiers from healthy soldiers by variation of antennal length and pronotal width. As shown in Figure 3, both minor and major soldiers were parasitized. However, none of the mature workers or L3 and L4 stages was parasitized.

**Figure 4.**
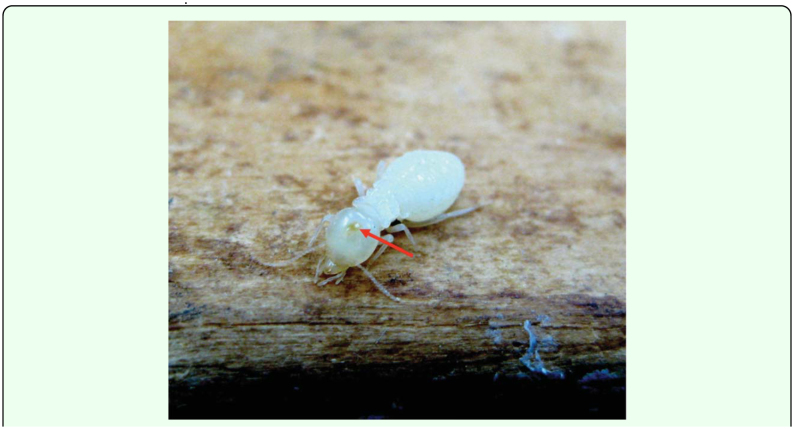
Parasitized *Macrotermes carbonarius* presoldier. A tiny dot (mouth hook of a larval parasitoid) can be seen inside the head capsule of the pre-soldier. High quality figures are available online.

In all instances, the larva was observed to bend its body to fill the entire head capsule of the parasitized soldier. The contents of each parasitized soldier's head capsule were totally consumed and empty after the departure of the larva.

In the present study, 16 parasitized presoldiers (Figure 4) with typically poor sclerotization were found. Under the microscope, the mouth hook of the larval parasitoid can be seen (as a tiny dot) inside the head capsule of the pre-soldier. The fly larvae in parasitized pre-soldiers were between 1.31 and 1.47 mm in length compared to 6.78–7.05 mm for fully developed larval parasitoids in mature soldiers.

## Discussion

The association between flies and termites has been known for a long time. Documentation of dipteran parasitism in *Macrotermes* is available for *M. gilvus* (Silvestri 1926; Schmitz 1938; Disney and Darlington 2000; Neoh and Lee 2009b; Neoh and Lee 2010), *Macrotermes subhyalinus* Rambur (Geigy et al. 1964), *Macrotermes malaccensis* (Havilandi) (Kemner 1925), and soldiers of *Macrotermes barneyi* Light (Sze et al. 2008). *M. carbonarius* can now be added to the list of hosts. We also demonstrate in the current study, for the first time, the morphological and biological changes that occur in *M. carbonarius* soldiers parasitized by *V. fasciventris*.

The most discernible morphological changes in the host termite are: (1) a decrease in total size of the parasitized major soldier; (2) a shortening of the mandibles; and (3) a change in the shape and size of the head capsule. The head capsule of parasitized soldiers appeared to be broader rather than longer compared to healthy soldiers. On the other hand, parasitized minor soldiers had a larger head capsule when compared to normal minor soldiers to allow room for the development of the larval parasitoid. In addition, parasitism induced a change in the antennal structure of *M. carbonarius* soldiers. Thapa (1981) also documented antennal alterations in parasitized soldiers of *M. malaccensis*. Thapa found that some parasitized soldiers had 18 segments and others had 19 segments. Brodeur and Boivin (2004) concluded that the host-parasitoid interaction may cause nutritional requirement stresses, which in turn alter host morphology.

Although parasitized soldiers were less aggressive than healthy soldiers when confronting *M. gilvus*, they still exhibited aggressive behavior and continued with routine activities (e.g. patrolling with workers and defense). However, Sze et al. (2008) reported the absence of aggressive behavior in parasitized *M. barneyi* soldiers. These authors also reported that parasitized *M. barneyi* soldiers displayed a series of larval preemergence behaviors that were not observed in the present study. Changes in host behavior in response to parasitism have been reported in other organisms such as bumblebees (Müller 1994), wooly bear caterpillars (English-Loeb et al. 1993), and tobacco hornworms (Thompson and Redak 2008), and are adaptive strategies that can help ensure the parasitoid's survival (Feener and Brown 1997). In the present study, the dipteran parasitoid consumed the entire contents of the head capsule and caused minor behavioral alterations to the host, *M. carbonarius*. Intriguingly, the empty-headed parasitized soldiers remained alive for 1–2 days after the larva departed.

The results of the present study clearly indicate that both minor and major soldiers are the targets of parasitism by *V. fasciventris*. Sze et al. (2008) also reported parasite infection in both soldier castes of *M. barneyi*. Although the head capsule may be distinctive in determining parasitoid infection as pointed out by Sze et al. (2008), in this study more features (e.g. pronotum width, length of third hind tibia, antennal length) were measured to obtain a better system of identifying parasitized individuals.

The adult female of *Verticia* spp. possesses a non-piercing ovipositor that is equipped with strong directed bristles (Sze et al. 2008). The existence of parasitized pre-soldiers may rule out the possible involvement of extensive physical contact of an adult female fly with the mature soldier castes. The results of the present study strengthen Sze et al.'s (2008) speculation that parasitism begins in the immature termite stages. Although none of the examined termite larvae (L3 and L4) contained fly larvae, we predict that the immature termite larvae from which the minor and major soldiers develop via a pre-soldier stage would be the first targets for parasitization. If true, the fly larva would complete its larval cycle using at least three termite developmental stages. This notion is further supported by the presence of a larval parasitoid, *Misotermes mindeni* Disney & Neoh (see Disney et al. 2009 for fly description), in the precursor pre-soldier stage (L4) of *M. gilvus* (Neoh and Lee, 2010). However, we failed to determine the termite stage in which the parasitism first occurred.

To date, the mechanism by which the dipteran parasitoid enters the termite host is unknown. However, there are several possible mechanisms: (1) the fly may enter the nest to deposit its eggs, as several genera of this subfamily were reported to oviposit in soil (*Ochromiya*, Altson 1932). Fly larvae may develop in the termite fungus comb (*Hemigymnochaeta*, Zumpt 1953) and food Stores (*Termitocalliphora*, Zumpt 1973), or the female adult fly may directly contact the hosts within a termite mound. This can only occur in breached nests, which would allow access into the mound's interior. (2) The fly may contact the workers and soldiers during open ground foraging excursions, and the workers and soldiers subsequently may transfer the eggs to the host through grooming. If the second scenario is true, one important question remains: How do fly eggs or larvae end up specifically in pre-soldiers? Both of these hypotheses lack empirical data and warrant further investigation.
